# Prediction of Sudden Cardiac Death Risk with a Support Vector Machine Based on Heart Rate Variability and Heartprint Indices

**DOI:** 10.3390/s20195483

**Published:** 2020-09-25

**Authors:** Marisol Martinez-Alanis, Erik Bojorges-Valdez, Niels Wessel, Claudia Lerma

**Affiliations:** 1 Facultad de Ingeniería, Universidad Anáhuac México, Huixquilucan 52786, Estado de Mexico, Mexico; marisol.martinez2@anahuac.mx; 2 Departamento de Estudios en Ingeniería para la Innovación, Universidad Iberoamericana Ciudad de México, Ciudad de México 01219, Mexico; erik.bojorges@ibero.mx; 3 Department of Physics, Humboldt-Universität zu Berlin, 10099 Berlin, Germany; wessel@physik.hu-berlin.de; 4 Departamento de Instrumentación Electromecánica, Instituto Nacional de Cardiología Ignacio Chávez, Ciudad de México 14089, Mexico

**Keywords:** sudden cardiac death, heart rate variability, heartprint, support vector machine

## Abstract

Most methods for sudden cardiac death (SCD) prediction require long-term (24 h) electrocardiogram recordings to measure heart rate variability (HRV) indices or premature ventricular complex indices (with the heartprint method). This work aimed to identify the best combinations of HRV and heartprint indices for predicting SCD based on short-term recordings (1000 heartbeats) through a support vector machine (SVM). Eleven HRV indices and five heartprint indices were measured in 135 pairs of recordings (one before an SCD episode and another without SCD as control). SVMs (defined with a radial basis function kernel with hyperparameter optimization) were trained with this dataset to identify the 13 best combinations of indices systematically. Through 10-fold cross-validation, the best area under the curve (AUC) value as a function of γ (gamma) and cost was identified. The predictive value of the identified combinations had AUCs between 0.80 and 0.86 and accuracies between 80 and 86%. Further SVM performance tests on a different dataset of 68 recordings (33 before SCD and 35 as control) showed AUC = 0.68 and accuracy = 67% for the best combination. The developed SVM may be useful for preventing imminent SCD through early warning based on electrocardiogram (ECG) or heart rate monitoring.

## 1. Introduction

**Figure 1:**
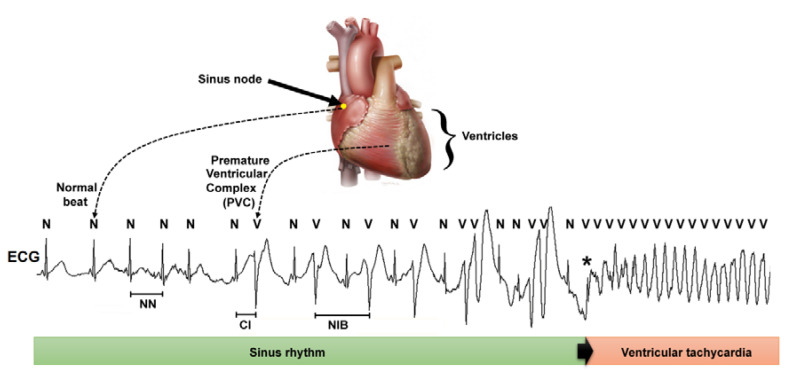
Schematic representation of the heart and a recording of cardiac electrical activity or an electrocardiogram (ECG) during an event of sudden cardiac death due to a transition from normal rhythm (sinus rhythm) to an episode of ventricular tachycardia, which was initiated by a premature ventricular complex or premature ventricular complex (PVC) (indicated by *). The heart rate variability (HRV) indices are based on the interval between consecutive normal beats (NN interval). By contrast, the heartprint indices include the coupling interval (CI) and the number of sinus intervening beats (NIB) between two consecutive PVCs.

Sudden cardiac death (SCD) refers to death from a cardiac cause within 1 h of symptom onset or during sleep in a patient who was previously stable [1]. In most cases, SCD is initiated by a transition from normal sinus rhythm to ventricular tachycardia (VT) or ventricular fibrillation (VF) that leads to asystole (Figure 1) [2]. Since SCD is preventable in those wearing an implantable cardioverter-defibrillator (ICD), identifying those that may benefit from an ICD is a great challenge, which remains elusive despite the many current risk-stratification tools [1,2,3].

Heart rate variability (HRV) refers to the time series of the fluctuations in the beat-to-beat heart period [4]. HRV is often obtained from the electrocardiogram (ECG) where the QRS complex of each beat is identified, and the time interval between consecutive beats (RR interval) is measured. HRV comprises only RR intervals between normal beats (NN intervals) (Figure 1). Most research on SCD prediction based on HRV indices has focused on SCD events that occur within long-term follow-up intervals (at least one year) and used HRV indices obtained from 24 h recordings (with time series of approximately 100,000 beats) [5,6]. Few studies have tested the prediction of SCD events within long-term follow-up intervals using HRV indices measured from 15 min recordings (with time series of about 1000 beats) [7,8]. Other studies have documented the predictive value of HRV indices for identifying SCD events in the short term (within the 72 h after arriving in the emergency room) [9] or imminent SCD (within the following 15 min in patients with HRV monitored by Holter recordings or ICDs) [10,11].

Since the HRV time series involve only heartbeats arising from the sinus node (the normal pacemaker of the heart), HRV indices do not include information about ectopic beats that arise from the ventricles, which are called premature ventricular complexes (PVCs). The occurrence of frequent PVCs increases the risk of SCD [12], and usually, one or more PVCs trigger an episode of VT or VF that leads to SCD [2]. The heartprint is a method developed for the quantitative analysis of different beat-to-beat intervals that involve normal sinus beats (labeled as N) and PVCs (labeled as V), including the coupling interval (CI) and the number of intervening beats between two PVCs (NIB) (Figure 1) [13]. Based on 24 h-length recordings, heartprint analysis has shown that having repeating forms of PVCs and low CI variability are risk markers for SCD in the long period of 2 years [14]. Additionally, having repeated forms of PVCs and short mean CI values predicts imminent SCD [11].

Beyond the traditional statistical methods for assessing the predictive value of HRV indices [5,7,8] and heartprint indices [11,14], different classification strategies have been proposed to predict SCD based on HRV indices, including support vector machines (SVM) [9,10] and artificial neural networks [15]. However, the studies based on different classifiers included only HRV indices and were tested on small datasets. In the present study, the aim was to develop an SVM for the prediction of imminent SCD based on combinations of both HRV and heartprint indices. Different combinations of indices were tested systematically to find an optimal combination that achieved the best performance for SCD prediction.

## 2. Materials and Methods

### 2.1. Data

**Figure 2:**
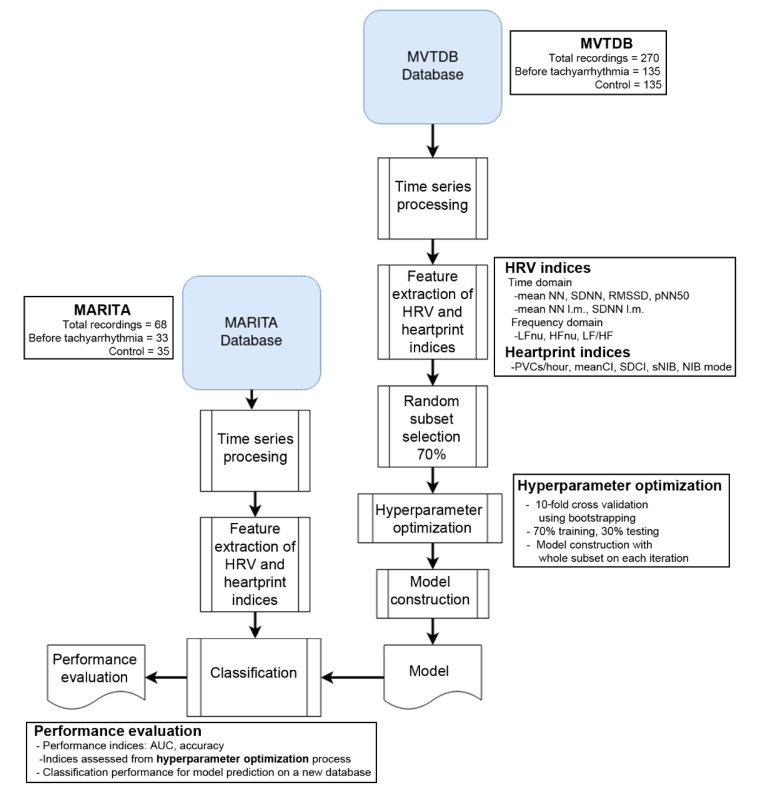
Flow chart of the study design: database description, a short description of the used features, structure of the classification method and performance evaluation. MARITA = MARITA study (Multivariate Analysis of RR-Intervals to predict ventricular TachyArrhythmia), MVTDB = Spontaneous Ventricular Tachyarrhythmia Database.

In this study, two datasets of RR interval time series were used: one dataset was used to train the SVM, while the other dataset was used to test the performance. This procedure guarantees the evaluation of classification models on completely unseen data, and it is a novelty regarding previous work (Figure 2).

The training dataset was obtained from the Spontaneous Ventricular Tachyarrhythmia Database (MVTDB, available at https://physionet.org/content/mvtdb/1.0/) [16]. This dataset includes 135 pairs of RR-interval time series obtained from electrograms recorded with ICDs (Medtronic Jewel Plus TM ICD 7218), each pair consisting of one recording ended before a VT or VF episode and another recording obtained during the follow-up visit (control recording). These recordings were obtained from 78 patients, whose clinical characteristics are shown in Table 1.

The second dataset was obtained from the MARITA study (Multivariate Analysis of RR-Intervals to predict ventricular TachyArrhythmia) [17]. The MARITA data of this study are available from the corresponding author on request. Sixty-eight RR-interval recordings were obtained from electrograms recorded with ICDs (Biotronik GmbH & Co, Berlin, Germany, model Biotronik Belos or microPhylax) from 13 patients (Table 1). From the sixty-eight recordings, thirty-three were taken before a tachyarrhythmia episode and thirty-five were used as control.

Compared to those in the MARITA dataset, the patients in the MVTDB dataset had lower left ventricular ejection fractions and less use of beta-blockers or other medications (Table 1). All other characteristics of the patients had no significant differences between the datasets.

### 2.2. Time Series Processing

Each of the recordings in both datasets contains the information of the RR-interval duration in seconds and the type of beat corresponding to each interval. If the beat was of sinus origin, it is marked with an “N” (normal beat), while the beats originated by a PVC are marked with a “V” (ventricular beat). The beat classification was based on the application of an adaptive filtering technique [18], which was revised manually by an expert to ensure correct beat classification. Additionally, the same filtering technique was used to replace the RR intervals from PVCs with estimated NN intervals. This provided an additional time series of only NN intervals for each recording (HRV time series).

Once all the time series were obtained, it was necessary to ensure all the recordings from both datasets had the same characteristics. Both datasets were checked to ensure all the recordings contained no PVCs at the end of the recording, since these could be an indicator of the start of the tachyarrhythmia episode, biasing the classifier with a false performance enhancement. Another important characteristic of the training dataset was that the duration of the recordings was around 1000 beats (on average, 15 min), while in the test dataset, the recordings were much longer. Therefore, the test dataset recordings were truncated to include the last 1000 beats only. By doing so, all the recordings from both datasets included the last 1000 beats before the occurrence of a tachyarrhythmia episode or 1000 beats from control recordings.

### 2.3. Feature Extraction of HRV and Heartprint Indices

HRV indices (based on time series analysis derived from NN intervals only) were calculated according to international recommendations [4], including the following time-domain indices: the average of the NN intervals (meanNN), the standard deviation of the NN intervals (SDNN), the root-mean-squared successive differences between consecutive beats (RMSSD), and the percentage of beats with differences greater than 50 ms between consecutive beats (pNN50). Additionally, the frequency-domain indices were estimated: the mean power within the low-frequency band (LF, 0.04 to 0.15 Hz), mean power within the high-frequency band (HF, 0.15 to 0.4 Hz), LF in normalized units (LFnu), HF in normalized units (HFnu) and LF/HF ratio. Power spectral analysis was performed using Welch’s periodogram method with a Hanning window of 300 data points with an overlap of 50%; NN time series were interpolated to achieve three samples per second, and linear trends were eliminated before the power spectral estimation. These HRV indices were estimated in 5 min-length segments, and an average was calculated from all the segments in each recording. Additionally, the average of the NN intervals in the last minutes of the recording (meanNN l.m.) and the standard deviation of the NN intervals in the last minutes of the recordings (SDNN l.m.) were measured [11,19].

The heartprint of each recording was obtained according to the method described in previous work [11,19,20]. The heartprint indices estimated were the number of PVCs/hour, mean coupling interval (meanCI), standard deviation of the coupling interval (SDCI), number of intervening sinus beats between consecutive PVCs (NIB) and most frequent NIB number (also called the NIB score or SNIB).

Prior to machine-learning modeling, all the HRV and heartprint indices were transformed to a linear scale within the range (0–1) by subtracting the minimum and dividing by the maximum value. The aim of this process was to avoid bias from indices with larger numerical ranges in their original scales. The same transformation factor was used in the training and performance tests. This could produce values for the tests outside the 0-to-1 range but did not bias the machine-learning approach to lead to erroneous results and is a better simulation for the behavior for online detection.

### 2.4. Classification

For the classification task, SVMs with the Radial Basis Function (RBF) kernel (Equation (1)) [21] were tuned by hyperparameter optimization. The RBF kernel has shown better performance compared to others [22,23,24]. This algorithm (SVM) was used because of the relatively low number of samples, which did not allow the use of a deep-learning approach in this study.

K(*x*_i_, *x*_j_) = exp(−γ ‖*x*_i_ − *x*_j_‖^2), γ > 0 (1)

The RBF kernel (K) was selected since it is a Gaussian function that maps the original feature map to a nonlinear space [25], based on data distribution. The γ (gamma) parameter is used to control the smoothness of the border, and *x*_i_, *x*_j_ refer to the data points (Equation (2)):

(2) $$y=\mathrm{sign}\left(\sum_{i}K\left(\omega_{i},\;x\right)+C\right)$$

where *y* is the label of any data point (*x*) to be classified, the learning process is referenced to find those w vectors (support vectors) that describe the border or frontier decision, and C refers to the cost.

In addition, it is necessary to select the indices that will be included in the SVM training. There were 16 available indices: 11 HRV indices and 5 heartprint indices, as described in Section 2.3. In order to perform the index selection for the SVM, a greedy search was performed, where different combinations of indices were tested to find the combination that produced the best results (based on the cross-validation criteria described below). The process started by testing every possible combination of two indices. Once the best combination of two indices was found, a third index was added. This process continued until all possible combinations, with a maximum of 16 indices, were tested. The combination with the best overall performance was selected for the SVM training.

To select the optimum values for both cost and the γ parameter, it was also necessary to conduct an exhaustive search. Hyperparameter optimization was performed by using a non-linear grid. For cost, a range from 0.5 to 10 in increments of 0.5 was used, while for γ, a range from 2^−15^ to 2^3^, with an incremental exponent increase of 0.5, was used. In order to improve the computational time, the selection of the best combination of indices was combined with the selection of the optimal values for cost and γ. The complete process was performed using a 10-fold cross-validation.

The training data were separated into 10 groups using the following procedure: for each patient, a pair or recordings was selected—one control recording and one recording preceding a tachyarrhythmia episode. This selection reduces the classification bias and ensures that more than one subject is used on each model. Once these recordings were selected, the patients were randomly distributed across the 10 groups. Both recordings corresponding to a patient were included in the group. As a result, every group had the same numbers of control recordings and of recordings preceding tachyarrhythmia. This procedure fixes the class prevalence and random threshold to 0.5, and the specificity and sensitivity levels to the same value.

Once the groups were obtained, the cross-validation was repeated several times. For each possible combination of indices, the optimum values for C and γ were obtained using a cross validation. Once these values were obtained for every combination of indices, the combination that produced the best values in the cross-validation process was selected.

After defining the optimum hyperparameters for the kernel function and for the index combination, the SVM was trained using all the records from the MVTDB dataset. The adjusted values for the selected indices were included, along with the outcome for each record (either ventricular tachyarrhythmia or control). The SVM obtained from this training process was then used on the MARITA dataset records for performance testing.

### 2.5. Statistical Analysis

Nominal variables are described as absolute values and percentages and were compared between groups by chi-squared tests or Fisher’s exact test. For continuous variables, the normality of distribution was tested by a Kolmogorov–Smirnov test. Variables with normal distributions are described as means and standard deviations and were compared by Student’s t-tests for paired or independent samples. Otherwise, these variables are described as medians (25th percentile–75th percentile) and were compared by Mann–Whitney U tests or Wilcoxon signed-rank tests. The effectiveness of the classifications was evaluated by receiver operator characteristic (ROC) curve analysis and the estimation of the area under the curve (AUC) for each index and the combinations of indices. The statistical analysis was performed with the software SPSS version 21 (IBM Corp, Armonk, NY, USA). The software MATLAB (The MathWorks Inc., Natick, MA, USA) and the library LibSVM (available at https://www.csie.ntu.edu.tw/~cjlin/libsvm/) were used for the validation and performance tests of the support vector machine. A *p*-value < 0.05 was considered as statistically significant.

## 3. Results

### 3.1. Conventional Statistical Analysis of Heart Rate Variability and Heartprint Indices

Table 2 shows the heart rate variability and heartprint indices compared by dataset (MARITA or MVTDB) and recording type (before VT/VF or control). In both datasets, the meanNN and meanNN in the last minute (l.m.) were shorter (i.e., heart rates were faster) in the recordings before VT/VF than in the control recordings (indicated by asterisks, *). Additionally, the recordings before VT/VF had larger LF/HF (i.e., larger sympathetic hyperactivity) and more PVCs/hour than control recordings (in both datasets). Only in the MVTDB dataset did the recordings before VT/VF show larger SDNN l.m. and shorter meanCI compared to the control recordings. In recordings before VT/VF, there were no significant differences between datasets, except for a larger LF/HF and smaller SDNN l.m. in the MARITA dataset compared to MVTDB (symbol ^&^). By contrast, in the control recordings, there were several differences between the datasets: the MVTDB had a larger LFnu and LF/HF, and smaller HFnu, meanNN l.m. and meanCI.

### 3.2. Definition and Validation of the Support Vector Machine

**Figure 3:**
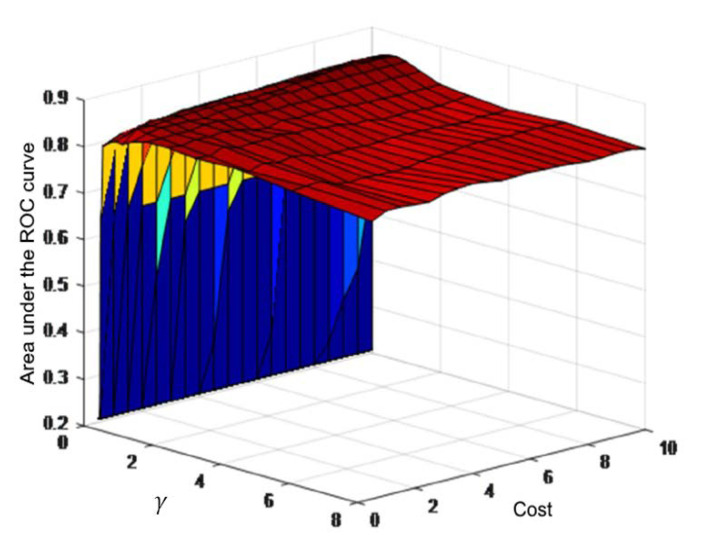
Example of the cross-validation process for a combination of 5 indices: meanNN l.m., SDCI, meanNN, meanCI and LF/HF. The area under the receiver operating characteristic (ROC) curve results are shown for the different values of cost and γ. The best area under the curve (AUC) value (0.8578) corresponds to a C value of 8.5 and a γ value of 0.7071.

An exhaustive search was carried out to define the best combination of the indices cost and γ’s values by a 10-fold cross-validation. In each validation, the area under the ROC curve (AUC) was calculated and the combination that achieved the highest AUC value was selected. Figure 3 shows an example of the cross-validation process and the corresponding results for a combination of five indices.

Table 3 shows the combinations of indices tested in the support vector machine. These indices were a combination of both HRV and heartprint characteristics. The selected HRV indices were meanNN, SDNN, RMSsd, pNN50, LFnu, HFnu and LF/HF. The values for meanNN and SDNN in the last minute before the tachyarrhythmia episode were also considered (meanNN l.m. and SDNN l.m.). The selected heartprint indices were PVCs/hour, meanCI, SDCI, sNIB and NIBmax.

The results of the cross validation for the support vector machine definition are shown in Table 4. This table shows the best results for each combination, from 2 to 14 indices. Each combination shows the average AUC values for the 10 folds of the cross-validation, along with the accuracy and the obtained values for cost and γ. A comparison of the AUC values for the different combinations of indices was carried out; however, there was no statistical significance between the different combinations. As a result, the combinations of four and five indices were selected since they presented, respectively, the highest accuracy and the highest AUC value.

### 3.3. Support Vector Machine Training and Performance Tests

Based on the selected characteristics for the SVM, two training processes were performed, one for the four-indices and one for the five-indices combination (Table 5). For the training stage, all the records of the MVTDB dataset were used to obtain the final SVM model, compared to the 10-fold cross validation where only a selected number of records was used. After obtaining this final model, the performance of the SVM was tested on the MARITA dataset. A lower performance was observed in the testing phase compared to the training phase, which was expected due to different methodological factors, as discussed below.

## 4. Discussion

The main contribution of this work is showing that combining HRV and heartprint indices through a support vector machine allows the identification of an increased risk of an imminent tachyarrhythmia episode in patients wearing an ICD. Specifically, combining heartprint indices such as sdCI and meanCI with HRV indices such as meanNN (both the average and in the last minute before a tachyarrhythmia episode) and LF/HF can be used for potential risk identification. Previous work based on conventional statistical analyses evaluated the predictive value for tachyarrhythmia of several HRV indices [15,19,26,27] and heartprint indices [14,19]. Among the HRV indices, the one reported with the best prognostic value for a tachyarrhythmia is a shorter meanNN (i.e., a faster heart rate) [27,28]. The grid search for the best combinations of indices confirmed the importance of such a simple feature, since the meanNN in the last minute and the averaged meanNN were among the three indices with the best predictive values. Moreover, having an increased heart rate may be associated with chronic or transient sympathetic nervous hyperactivity before a tachyarrhythmia episode [29]. Notably, another HRV index (LF/HF) that is also associated with higher sympathetic nervous activity prior to some forms of VT [30] was included in the combinations with higher predictive values. Notably, these indices that are compatible with a scenario of higher sympathetic hyperactivity were significantly different before VT/VF than in the control recordings in both datasets (Table 2), despite the differences in treatment with beta-blockers (i.e., drugs that decrease the effect of sympathetic nervous activity on the heart); sympathetic hyperactivity was more frequent in the patients of the MARITA dataset than in those of the MVTDB dataset (Table 1). Nevertheless, the physiological interpretation of LH/HF has to be considered with caution, as it has been debated in the literature for a long time [31].

Regarding the heartprint indices, the ones involved in the best performance combinations are related to the CI, having either high variability of the CI (i.e., a larger SDCI) or more premature PVCs (i.e., shorter meanCI). The heartprint index NIBmax, which is related to a high incidence of PVCs with a certain repetitive pattern, also appeared in some combinations with good predictive performance. These results agree with previous conventional statistical analyses [11,14]. Our results are the first report that considered combining heartprint with HRV indices in a machine-learning algorithm to predict SCD.

The approach of support vector machines has been used previously for arrhythmia identification based on data derived from ECG [32,33,34,35]. Some of these recordings were obtained from hospitalized patients, while others were obtained from 24 h ambulatory Holter recordings. All these previous studies used long-term recordings, in contrast with the present work, which focused on the analysis of short-term recordings (~1000 heartbeats). Our results show that several combinations of indices had outstanding performance for predicting imminent tachyarrhythmia. These indices obtained from short-term recordings could be tested as predictors of tachyarrhythmia and other cardiac events using implantable cardiac monitors [36], as predictors of mortality in patients in the intensive care unit [34], and as predictors of a heart attack in patients in the emergency room [9]. Nevertheless, the support vector machine was tested exclusively on data obtained from patients with a high risk of SCD who were wearing an ICD. It is necessary to extend this analysis to data from the general population with an unknown risk of SCD and to assess the impact of comorbidities on the use of both HRV and heartprint indices as risk markers for SCD.

In the present work, the heartbeat classification (either as normal or PVC) was obtained from RR intervals with a previously validated algorithm that is highly efficient in most cases [19]. Such an algorithm is less efficient in RR-interval time series with a high number of complex PVCs, and heartbeat classification had to be corrected manually [11,19]. However, having an ECG signal is optimal for ensuring proper beat classification, even when using an automatic detection and classification method, through direct comparison against the ECG signal. Further work is needed to test the support vector machine proposed in the present study with data where proper beat classification was verified against the ECG signal.

The selection of the indices used for training and testing with the support vector machine involved no preprocessing of the data (e.g., transforming the scale of some indices). The selection of the indices was based solely on their performance in the grid search and cross validation on the training dataset. In a future exploration, it will be interesting to evaluate the effect of preprocessing the data to improve the performance of the vector support machine. Such exploration requires a new analysis of the data where the characteristics of the indices are checked to choose the adequate processing that would effectively improve the predictive capacity of the vector support machine.

Regarding the performance of the final SVM model, which was lower in the testing stage compared to the training stage, such a difference is expected due to the use of a testing dataset that proceeds from a different population than that of the training dataset. Machine-learning problems focus on the search for target functions that are able to correctly predict outcomes with a different testing dataset that is not necessarily similar to the training dataset [37]. Notably, previous studies of machine-learning methods for predicting imminent SCD events did not test their SVM models with different training datasets but used other sampling strategies from the same training dataset [9,10,15,38,39]. The lower performance achieved using a completely unseen dataset could be associated with an overfitting caused by the normalization procedure, and it could be desirable to test other normalization techniques such as elastic nets or standardization. Nevertheless, by using a different testing dataset, the SVM model presented in this work was shown to demonstrate a series of indices that are useful in the detection of an imminent tachyarrhythmia episode.

Future work can include the use of other classification methods, such as neural networks, random forest, genetic algorithms, and deep-learning or hidden-Markov models, to compare their individual or combined performance in predicting tachyarrhythmia [22,23,24,40,41,42]. Additionally, other indices (for instance, those derived from the non-linear analysis of HRV) could be explored. It is also desirable to test the performance of these methodologies for risk classification in other types of patients with other types of devices, such as ambulatory patients with implantable cardiac monitors or hospitalized patients with ECG monitors.

## 5. Conclusions

The development of a support vector machine in this work showed combinations of both HRV and heartprint indices with outstanding predictive value for imminent tachyarrhythmia in patients wearing an ICD. These findings based on short-term recordings may be useful for preventing SCD through the addition of early warnings in settings with RR-interval monitoring such as intensive care units or the emergency room.

## Figures and Tables

**Table 1:** Clinical characteristics of patients and number of recordings per patient for both datasets. Data are shown as either absolute values (percentages) or medians (25th percentile–75th percentile). The statistical significance of the difference between databases is shown in the *p*-value column.

	MVTDB (N = 78)	MARITA (N = 13)	*p*-Value
Age (years)	62 (53–69)	62 (57–69)	0.669
Sex			0.312
Male	63	12	
Female	15	1	
Diagnosed cardiac disease			
Dilated cardiomyopathy	26 (33%)	1 (8%)	0.061
Ischemic cardiomyopathy	49 (63%)	11 (85%)	0.125
Left ventricular ejection fraction (%)	25 (20–35)	40 (33–45)	0.006
New York Heart Association class			0.068
I or II	65 (83%)	8 (62%)	
III or IV	13 (17%)	5 (38%)	
Medication			
Beta-blocker	27 (35%)	12 (92%)	<0.001
Digoxin	23 (29%)	5 (38%)	0.516
Antiarrhythmic drug	26 (33%)	4 (31%)	0.856
Others	9 (12%)	13 (100%)	<0.001
None	19 (24%)	0 (0%)	0.045
Number of recordings per patient			
Before tachyarrhythmia	1 (1–2)	3 (1–3)	0.059
Total before tachyarrhythmia	135	33	
Control	1 (1–2)	2 (1–3)	0.221
Total control	135	35	

**Table 2:** Heart rate variability and heartprint indices evaluated from recordings before tachyarrhythmia (ventricular tachycardia (VT)/ventricular fibrillation (VF)) or control recordings for both datasets. Data are shown as medians (25th percentile–75th percentile).

	MARITA		MVTDB	
	Before VT/VF (N = 33)	Control (N = 35)	Before VT/VF (N = 135)	Control (N = 135)
meanNN (ms)	705 (610–784)	759 (678–873) *	676 (607–819)	787 (707–898) *
SDNN (ms)	46 (28–71)	41 (30–78)	49 (34–77)	50 (27–78)
RMSSD (ms)	15 (11–24)	15 (12–20)	18 (13–31)	20 (13–35)
pNN50 (%)	2.00 (0.50–6.71)	1.00 (0.40–3.50)	0.49 (12.38–2.09)	2.75 (0.49–2.75)
LFnu	49.4 (42.2–63.2)	56.5 (52.7–67.5) ^&^	45.2 (33.5–56.2)	50.1 (39.3–60.6) *
HFnu	25.7 (20.3–32.5)	16.9 (12.1–32.5) ^&^	31.3 (21.5–42.9)	29.6 (20.0–38.7)
LF/HF	1.6 (1.0–3.1) ^&^	2.7 (2.0–3.9) *^,&^	1.2 (0.7–2.4)	1.6 (0.9–2.3) *
meanNN l.m. (ms)	625 (560–801)	764 (676–823) *^,&^	625 (532–734)	803 (726–954) *
SDNN l.m. (ms)	19 (13–35) ^&^	22 (15–31)	65 (39–90)	24 (13–38) *
PVCs/hour ^¶^	154 (63–500)	79 (11–181) *	159 (42–433)	71 (11–351) *
meanCI (ms) ^¶^	485 (444–522)	493 (446–553) ^&^	520 (446–592)	575 (509–636) *
SDCI (ms) ^¶^	41 (32–71)	59 (30–75)	63 (46–78)	57 (37–88)
sNIB ^¶^	14 (2–36)	4 (1–9)	9 (3–25)	5 (1–22)
NIB mode ^¶^	2 (1–3)	2 (1–8)	1 (0–4)	2 (0–7)

l.m.: last minute; * *p*-value < 0.05 (before VT/F vs. control); ^&^ *p*-value < 0.05 (MARITA vs. MVTDB); ^¶^ heartprint indices.

**Table 3:** Combination of indices for the support vector machine.

Combination	Indices
2 indices	meanNN l.m., SDCI *
3 indices	meanNN l.m., SDCI *, meanNN
4 indices	meanNN l.m., SDCI *, meanNN, meanCI *
5 indices	meanNN l.m., SDCI *, meanNN, meanCI *, LF/HF
6 indices	meanNN l.m., SDCI *, meanNN, meanCI *, LF/HF, SDNN l.m.
7 indices	meanNN l.m., SDCI *, meanNN, meanCI *, LF/HF, SDNN l.m., LFnu
8 indices	meanNN l.m., SDCI *, meanNN, meanCI *, LF/HF, SDNN l.m., LFnu, NIBmax *
9 indices	meanNN l.m., SDCI *, meanNN, meanCI *, LF/HF, SDNN l.m., LFnu, NIBmax *, RMSsd
10 indices	meanNN l.m., SDCI *, meanNN, meanCI *, LF/HF, SDNN l.m., LFnu, NIBmax *, RMSsd, HFnu
11 indices	meanNN l.m., SDCI *, meanNN, meanCI *, LF/HF, SDNN l.m., LFnu, NIBmax *, RMSsd, HFnu, sNIB *
12 indices	meanNN l.m., SDCI *, meanNN, meanCI *, LF/HF, SDNN l.m., LFnu, NIBmax *, RMSsd, HFnu, sNIB *, SDNN
13 indices	meanNN l.m., SDCI *, meanNN, meanCI *, LF/HF, SDNN l.m., LFnu, NIBmax *, RMSsd, HFnu, sNIB *, SDNN, PVCs/hour
14 indices	meanNN l.m., SDCI *, meanNN, meanCI *, LF/HF, SDNN l.m., LFnu, NIBmax *, RMSsd, HFnu, sNIB *, SDNN, PVCs/hour, pNN50

l.m.: last minute. * Heartprint indices.

**Table 4:** Results for the cross-validation for the parameter definition of the support vector machine. The area under the ROC curve and the accuracy are presented as mean ± standard deviation. The optimal values for cost (C) and gamma (γ) are also shown. The random threshold was set to 50 for all tests.

Combination	AUC	Accuracy	*C* Value	*r* Value
2 indices	0.8339 ± 0.0655	82.14 ± 8.18	7.5	0.1250
3 indices	0.8433 ± 0.0827	84.55 ± 7.25	9.0	1.0000
4 indices	0.8557 ± 0.0799	85.80 ± 6.71 ^&^	5.0	8.0000
5 indices	0.8578 ± 0.0768 *	84.64 ± 6.61	8.5	0.7071
6 indices	0.8403 ± 0.0716	85.27 ± 5.06	8.5	0.7071
7 indices	0.8397 ± 0.0811	84.02 ± 5.13	2.0	0.7071
8 indices	0.8534 ± 0.0700	83.75 ± 9.09	8.0	0.1250
9 indices	0.8379 ± 0.0671	81.34 ± 5.65	8.0	0.7071
10 indices	0.8421 ± 0.0708	82.77 ± 5.91	1.5	1.0000
11 indices	0.8378 ± 0.1001	84.20 ± 8.91	6.0	0.1768
12 indices	0.8271 ± 0.0841	81.25 ± 6.13	8.0	0.2500
13 indices	0.7972 ± 0.0705	79.55 ± 6.24	10.0	0.1250
14 indices	0.8022 ± 0.1060	80.27 ± 10.25	2.0	0.7071

AUC: Area under the ROC curve; * best AUC; ^&^ best accuracy.

**Table 5:** Results of the training and testing of the support vector machine. The area under the ROC curve and the accuracy are presented with their 95% confidence intervals.

	Training		Testing	
Combination	AUC	Accuracy	AUC	Accuracy
4 indices	0.892 (0.852–0.931)	82.593	0.678 (0.550–0.806)	67.647
5 indices	0.858 (0.812–0.903)	77.407	0.646 (0.515–0.777)	63.235

AUC: Area under the ROC curve.
